# Myocardial Infarction with Non-Obstructive Coronary Arteries (MINOCA): Focus on Coronary Microvascular Dysfunction and Genetic Susceptibility

**DOI:** 10.3390/jcm12103586

**Published:** 2023-05-21

**Authors:** Paolo Severino, Andrea D’Amato, Silvia Prosperi, Vincenzo Myftari, Lorenzo Colombo, Elisa Tomarelli, Alice Piccialuti, Gianluca Di Pietro, Lucia Ilaria Birtolo, Viviana Maestrini, Roberto Badagliacca, Gennaro Sardella, Francesco Fedele, Carmine Dario Vizza, Massimo Mancone

**Affiliations:** Department of Clinical, Internal, Anesthesiology and Cardiovascular Sciences, Sapienza University of Rome, Viale del Policlinico, 155, 00161 Rome, Italy; paolo.severino@uniroma1.it (P.S.); damatoandrea92@gmail.com (A.D.); silviapro@outlook.it (S.P.); vincenzo.myftari@gmail.com (V.M.); colombolorenzo90@yahoo.it (L.C.); elisa.tomarelli@yahoo.it (E.T.); alicepiaccialuti@gmail.com (A.P.); gianlucadipietro95@gmail.com (G.D.P.); ilariabirtolo@gmail.com (L.I.B.); viviana.maestrini@uniroma1.it (V.M.); roberto.badagliacca@uniroma1.it (R.B.); rino.sardella@uniroma1.it (G.S.); francesco.fedele@uniroma1.it (F.F.); dario.vizza@uniroma1.it (C.D.V.)

**Keywords:** MINOCA, INOCA, coronary microvascular dysfunction, coronary flow reserve, index of microvascular resistance, genetic predisposition

## Abstract

Among the most common causes of death worldwide, ischemic heart disease (IHD) is recognized to rank first. Even if atherosclerotic disease of the epicardial arteries is known as the leading cause of IHD, the presence of myocardial infarction with non-obstructive coronary artery disease (MINOCA) is increasingly recognized. Notwithstanding the increasing interest, MINOCA remains a puzzling clinical entity that can be classified by distinguishing different underlying mechanisms, which can be divided into atherosclerotic and non-atherosclerotic. In particular, coronary microvascular dysfunction (CMD), classifiable in non-atherosclerotic mechanisms, is a leading factor for the pathophysiology and prognosis of patients with MINOCA. Genetic susceptibility may have a role in primum movens in CMD. However, few results have been obtained for understanding the genetic mechanisms underlying CMD. Future studies are essential in order to find a deeper understanding of the role of multiple genetic variants in the genesis of microcirculation dysfunction. Progress in research would allow early identification of high-risk patients and the development of pharmacological, patient-tailored strategies. The aim of this review is to revise the pathophysiology and underlying mechanisms of MINOCA, focusing on CMD and actual knowledge about genetic predisposition to it.

## 1. Introduction

Among the most common causes of death worldwide, ischemic heart disease (IHD) is recognized to rank first, with increasing frequency despite diagnostic and therapeutic progresses [1]. Atherosclerotic disease involving the epicardial arteries is known as the leading cause of IHD [2]. However, among patients presenting an ST elevation myocardial infarction (STEMI), about 10% had no significant coronary artery disease (CAD) on angiography [3]. This demonstrates how other pathophysiological mechanisms may be involved. According to the Fourth Universal Definition of Myocardial Infarction [4], the combination of symptoms and a positive cardiac biomarker, cardiac troponin, is diagnostic of acute myocardial injury (AMI). When coronary plaques ≥50% of epicardial vessels diameter are excluded at coronary angiography, and other alternative diagnoses can be ruled out (i.e., pulmonary embolism, myocarditis), the diagnosis of myocardial infarction with non-obstructive coronary artery disease (MINOCA) can be applied [4,5,6]. MINOCA was documented for the first time more than 75 years ago, and since then, it has been the object of several clinical studies. These latter have reported a prevalence of MINOCA from 5% to 6% of AMI cases, with a range between 5% and 15% depending on the population examined [7]. The COAPT study reported MINOCA in 5.8% of patients with myocardial infarction [8]; in the GENESIS-PRAXY trial, MINOCA was identified in 8.2% of the sample examined [9]. 

Another definition worth mentioning is myocardial ischemia with non-obstructive coronary arteries (INOCA). This is an entity that has been found to be much more prevalent in women, associated with the increased risk of major adverse cardiovascular events (MACE), heart failure with preserved ejection fraction (HFpEF), coronary microvascular dysfunction (CMD), and stroke. The major difference between MINOCA and INOCA is that the former requires evidence of acute myocardial infarction and, at the same time, angiographic proof of non-occlusive coronary disease. Risk factors predisposing to these two conditions also differ: those for MINOCA include arterial hypertension, psychological stress, younger age, and platelet disorders, while risk factors for INOCA relate to older age, compared to patients affected by MINOCA, and traditional cardiovascular risk factors such as dyslipidemia, diabetes mellitus, and smoking [10].

Strong evidence supports the role of traditional risk factors in the pathogenesis of the disease [11,12], which have also been associated with a poor prognosis [13]. In particular, chronic hyperglycemia is associated with a significantly reduced endothelial-dependent and endothelial-independent coronary vasodilator function [14]. In addition, chronic inflammation is frequently observed in patients with frequent ischemic episodes, because it promotes an overproduction and storage of cellular reactive oxygen species (ROS) [15]. 

Notwithstanding the increasing interest, MINOCA remains a puzzling clinical entity, also because of the discrepancy between the population enrolled in clinical studies and the population affected by it in clinical practice. Indeed, even if epidemiologically younger women are the most affected, the vast majority of studies enroll men and older patients, biasing our actual knowledge [16]. Lastly, patients with MINOCA have a variable prognosis that is mainly dependent on the underlying cause [17]. 

This review aims to explore the state of the art of this disease with a particular focus on the pathophysiological mechanism of CMD. 

## 2. Pathophysiology and Underlying Mechanisms of MINOCA

### 2.1. Coronary Physiology and Regulation of Coronary Blood Flow

The coronary arterial system consists of a vessel network characterized by different sizes and functions. On the one hand, epicardial coronary arteries have minimal resistance to coronary flow, and their tone is mainly regulated by shear stress and endothelial function. On the other hand, arterioles represent most of the resistance to the heart circuit and adjust their blood distribution according to the needs of myocardial tissue metabolism. Pre-arterioles, arterioles, and capillaries represent the coronary microcirculation [18].

Even if the real role of the systems responsible for the cross-talk between coronary flow and myocardial metabolism has not been clearly identified, it is known that, in physiological conditions, coronary blood flow (CBF) regulation is mediated by various different systems, including endothelial, neurohumoral, nervous, metabolic, and myogenic mechanisms [19,20]. Firstly, endothelial cells affect vasomotor activity through vasoactive substances, such as vasodilator nitric oxide (NO) and vasoconstrictor endothelin-1 (ET-1), whose balance may be altered by oxidative stress and inflammation. For instance, ROS augmentation can foster the transformation of NO into peroxynitrite radicals, switching the endothelial NO synthetase (eNOS) from a NO- to a ROS-producing enzyme. It causes a reduction in NO-mediated vasodilation and increases ET-1 vasoconstriction activity through the RhoA/Rho-kinase pathway [21,22]. Secondly, the resistance to the coronary vascular network is regulated by the parasympathetic nervous system and the synthesis of acetylcholine, which causes vasodilation through the production of NO by eNOS. The sympathetic system acts as a vasoconstrictor, through the α-receptors expressed on the epicardial vessels, and as a vasodilator, through the β-receptors of the intramyocardial vessels and the Ca^++^-activated K^+^ (KCa) channels [23,24]. In addition, myogenic mechanisms enable coronary self-regulation systems that maintain the blood flow constant, despite changes in the perfusion pressure. The increase in myogenic tone is due to Ca^++^-dependent signals through the L-type Ca^++^ channels and the voltage-gated K^+^ (Kv) channels [24]. Moreover, metabolic regulation has a crucial role in working through adenosine, adenosine triphosphate (ATP), adenosine diphosphate (ADP), prostaglandins, and ROS, causing the dilation of arterioles. In normal conditions, creatine kinase inhibits adenylate kinase, and through inhibition by ATP, the ATP-sensitive potassium channels (K-ATP) are mostly in a “closed” state. Hypoxia reduces the activity of creatine kinase and increases adenylate kinase activity with the consequent production of adenosine monophosphate (AMP), K-ATP channel opening, hyperpolarization of the membrane, and coronary vasodilation [25,26,27].

CBF regulation also depends on several ion channels activities, which are the end effectors of all the regulation mechanisms acting at the coronary level. They are expressed by endothelial cells and vascular smooth muscle cells (VSMCs). In particular, K-ATP channels, Kv channels, and voltage-gated Na^+^ (Nav) channels regulate the concentration of calcium in both coronary smooth muscle and endothelial cells, allowing the rapid response of both the endothelium and vascular smooth muscle layer to the continuously changing demands of the myocardium [28]. Thus, because of their role in repolarization in coronary vascular cells, vascular tone abnormalities are strictly connected to the imbalance of ion channel activity and expression [29]. Ion channels have a central role in the continuous cross-talk between coronary circulation and the myocardium, and their abnormalities contribute to CMD and IHD. 

### 2.2. MINOCA and Pathophysiological Mechanisms

**Figure 1:**
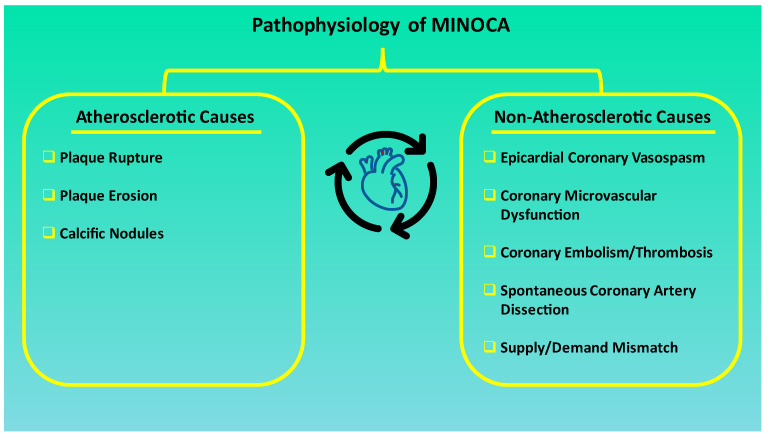
Summary of the pathophysiological mechanisms of MINOCA. Underlying pathophysiological mechanisms of MINOCA can be divided into atherosclerotic and non-atherosclerotic. MINOCA: Myocardial infarction with non-obstructive coronary artery disease.

MINOCA can be classified by distinguishing different underlying pathophysiological mechanisms, which can be divided into atherosclerotic and non-atherosclerotic, as illustrated in Figure 1.

Atherosclerotic causes essentially concern plaque disruption. This term encompasses plaque rupture, plaque erosion, and calcific nodules, which can then lead to thrombus formation, laying the ground for myocardial infarction, whose presence can be confirmed by intracoronary imaging. Approximately one-third of patients affected by MINOCA are found to have a plaque disruption detected on intravascular ultrasound (IVUS) [7]. 

The non-atherosclerotic causes most notably include epicardial coronary vasospasm, CMD, coronary embolism/thrombosis, spontaneous coronary artery dissection, and supply/demand mismatch [6]. Coronary artery spasm is due to vascular smooth muscle hyperreactivity in response to endogenous or exogenous (e.g., cocaine) vasospastic substances [5]. It has been reported that over a quarter of patients with MINOCA undergoing provocative testing are shown to have inducible spasm [17]. CMD can also contribute to the development of MINOCA. As a matter of fact, if epicardial coronary arteries can be easily visualized by coronary angiography and readily revascularized in case of obstructive atherosclerotic plaque presence, they constitute only the tip of the iceberg for what relates to coronary resistance when myocardial ischemia is present in the absence of CAD. Indeed, 70% of coronary resistance is provided by the coronary microcirculation [30]. Coronary thrombosis or embolism may be involved in the genesis of MINOCA as well, mostly by affecting the coronary circulation at the microvascular level. Embolism at the level of the branches of the epicardial coronary arteries may also represent the causative mechanism. Thrombosis may be due to inherited or acquired thrombotic disorders, whereas embolism may be due to coronary or systemic embolization, eventually lodging at coronary microcirculation [30]. Spontaneous coronary dissection usually causes MINOCA by means of luminal obstruction. However, particularly when occurring at the level of the coronary microcirculation, obstruction may not always be evident upon coronary angiography, hence leading to the diagnosis of MINOCA. This subtype tends to occur more frequently in younger and female subjects, and a link between hormones, pregnancy and delivery leading to changes in intima-media composition has been identified [5,30]. Interestingly, most of the dissections have been shown to occur in absence of atherosclerotic disease [5,30]. Supply/demand mismatch, the pathophysiological ground of the so-called type II myocardial infarction, is most commonly due to arrhythmias, hypotension, and hypoxia. As approximately half of patients experiencing such a mismatch are not shown to have a significant CAD, they may be classified as MINOCA [31].

In addition to these mechanisms, it is also worth mentioning Takotsubo syndrome (TTS) because it is still controversial if it should be classified within MINOCA or not. Its clinical presentation, indeed, is characterized by acute, reversible heart failure (HF) with myocardial stunning in the absence of occlusive CAD [32]. Its pathophysiological mechanism seems to depend on dysregulation in the sympathetic neurohormonal axis. This is usually consequent to a stressful event, which plays a vital role, leading to endothelial dysfunction and vasospasm [33]. Moreover, TTS shows clear differences from the rest of the MINOCA group: it presents a more aggressive clinical presentation and worse in-hospital outcomes with better long-term cardiovascular prognosis [34]. 

Moreover, it is worth mentioning that an increased prevalence of MINOCA has been found, compared to CAD-related myocardial infarction, in patients with active cancer. The neoplasm itself and the antineoplastic treatment may lead to coronary spasm, endothelium damage, and acute thrombosis, and the risk of myocardial infarction is higher when chemotherapy drugs of different groups are administered together. Interestingly, it has also been shown that patients with MINOCA are affected by myocardial injury on a lesser scale due to the prevalence of edema with small foci of necrosis [35].

Considering the high incidence of MINOCA/INOCA, whose detection is becoming increasingly feasible thanks to better awareness and diagnostic advancements in the field, there is an expanding interest in the pathophysiological basis of this complex entity. Such an interest is particularly powered by the willingness to more precisely outline its less evident and more underhanded mechanisms, which constitute a promising field of research yet to be fully elucidated. 

## 3. Coronary Microvascular Dysfunction in MINOCA

Coronary microcirculation consists of blood vessels smaller than 4–5 mm in diameter (i.e., pre-arterioles, arterioles, and capillaries), whose main function is the regulation of coronary vascular resistance. Of the total resistance, 70% is provided by the coronary microcirculation [7,36,37]. Coronary microcirculation is also a reservoir of myocardial blood; in fact, it contains almost 90% of total myocardial blood volume [38]. Coronary pre-arterioles have a diameter ranging from 500–400 μm to 100 μm, and their function is to maintain constant the pressure at the origin of arterioles into a limited range, despite variations in the epicardial coronary perfusion pressure and flow [36]. Coronary arterioles have a diameter ranging from 100 μm to 20–10 μm, and their function is to provide myocardial blood supply according to myocardial oxygen consumption; this is the reason why they are the main site of regulation of coronary vascular resistance [7,17,36,37,38]. Capillaries are made up of a single endothelial layer and a basal lamina with a diameter of approximately 10–12 μm, whose function is to ensure metabolic and respiratory exchanges [17]. The endothelium has a crucial role in cardiovascular homeostasis, in particular myocardial capillaries [39,40].

CMD could be defined as a set of dysfunctions affecting both the structure and function of coronary microcirculation, resulting in inadequate coronary blood supply [17,39,40,41]. CMD is a leading factor in the pathophysiology and prognosis of patients with INOCA and MINOCA. CMD is present in up to 50% of patients declaring one or more episodes of chest pain but without obstructive CAD on angiography, a condition that more commonly affects women and patients with multiple cardiovascular risk factors [42]. Recently, it has been estimated that approximately half of patients with non-obstructive CAD have CMD, especially females [43].

The prognostic significance of CMD has been evaluated by a coronary angiography-derived index of microvascular resistance (IMR). Patients with higher IMR had a higher rate of MACE (36.4%) as compared to patients with lower IMR (13%) [44]. Coronary angiography-derived IMR is a strong predictor of the clinical outcome in patients with MINOCA [44]. Severe CMD is demonstrated in patients with INOCA, and they have a higher risk of MACE [45]. CMD finds its roots in a variable combination of functional and structural mechanisms affecting coronary microcirculation [41]. According to literature data, the best-known factors are the following:

- Structural mechanisms, such as abnormal vascular remodeling [38,41,46], capillary rarefaction [47,48], luminal obstruction [5,40,49,50,51], vascular wall infiltration [41], and extrinsic vascular compression (e.g., edema, fat infiltration, amyloidosis, and perivascular fibrosis) [41,47,48].
- Functional mechanisms, such as endothelial dysfunction [40,49,52,53] and vascular smooth muscle cell hyperreactivity [17,41].

All these mechanisms lead to impaired vasodilatation and/or enhanced vasoconstriction.

**Figure 2:**
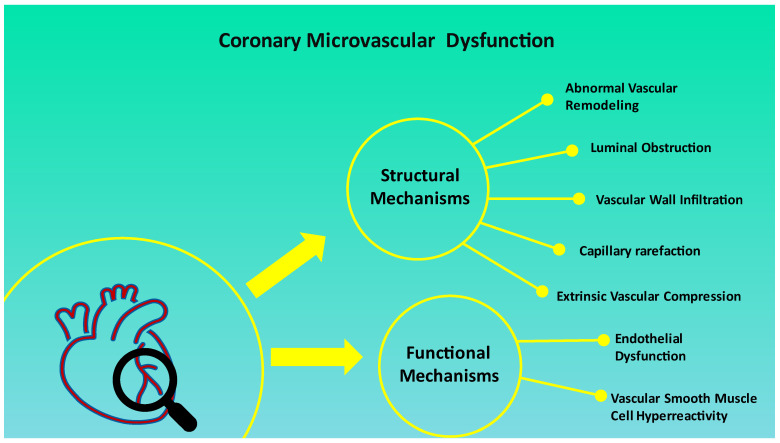
Structural and functional mechanisms of coronary microvascular dysfunction.

The impact of every single factor in determining CMD is not clearly defined. However, it is known that the combination of structural and functional mechanisms is interconnected, and it can result in inadequate CBF and myocardial damage [41]. The functional and structural mechanisms of CMD are summarized in Figure 2.

Abnormal vascular remodeling is mostly a chronic alteration of microcirculation vessel composition, characterized by changes in diameter and thickness, both with regard to the cellular compartment (VMSCs and endothelial cells) and the extracellular component [41]. Vascular remodeling is mostly caused by the thickening of the medial wall due to proliferated smooth muscle cells and perivascular fibrosis [46]. It generally induces a reduction in luminal size, which has a negative effect on CBF [38]. 

Capillary rarefaction and perivascular fibrosis are usually co-present in cardiac remodeling following myocardial infarction, pressure overload, or myocarditis [47]. Endothelial cells regulate vasodilatation by releasing in loco vasodilator substances, especially NO. NO protects the integrity of the endothelium and counteracts apoptosis, inhibiting fibrosis and platelet aggregation [39,54]. 

Impaired endothelial cells decrease the production of NO, resulting in collagen deposition, poor angiogenesis, endothelial-to-mesenchymal transition, and proliferation of fibroblasts (which increase the production of collagen and fibronectin). All of these aspects lead to capillary rarefaction (i.e., loss of perfused microvessels) and perivascular fibrosis [48]. It must be noted that fibroblast proliferation and the increasing production of extracellular matrix proteins raise the distance between capillaries and myocytes, exposing the myocardium to the risk of hypoxia, even more in the case of reduced blood flow. When arteriolar remodeling co-exists with decreased capillary density, the effect on blood flow reduction is more than additive [52]. 

Additionally, hormones and stress may have a role in MINOCA pathophysiology. It has been speculated that MINOCA incidence may be connected to circadian stress. For example, MINOCA occurs significantly less during weekends and holidays; however, this finding did not impact long-term mortality [55].

Currently, it has been demonstrated that MINOCA can be related to the luminal obliteration of coronary microcirculation due to thromboembolic mechanisms [5,49]. Coronary thromboembolism starting directly from the left heart is typically caused by atrial fibrillation, valvular disease, intraventricular thrombi, and cardiac tumors (e.g., myxoma and papillary fibroelastoma) [5,40]. Another relevant possibility is debris from non-critical epicardial atherosclerotic plaque following spontaneous or iatrogenic rupture. The release of debris and thrombogenic substances from atherosclerotic plaques may also be a chronic phenomenon, resulting in progressive CMD [50]. On the other hand, coronary thromboembolism can also be paradoxical in the presence of right–left shunt, such as in the instance of patent foramen ovale, atrial septal defect, and, rarely, arteriovenous fistula [5]. 

It is not uncommon that these patients have co-occurring thrombophilic disorders. Indeed, routine thrombophilic screening performed on patients with MINOCA showed a prevalence of about 14% of these disorders, either of inherited type or of acquired type, which is higher than in the general population [51,52,53,54,56]. Inherited thrombophilia mainly includes factor V Leiden (prevalent in 5% of general population), elevated factor VIII/von Willebrand factor (prevalent in 25% of general population), protein C deficiency, protein S deficiency, antithrombin III deficiency, and mutation of prothrombin G20210A [7]. Acquired thrombophilia mainly includes autoimmune antiphospholipid syndrome, heparin-induced thrombocytopenia, thrombotic thrombocytopenic purpura, and myeloproliferative disorders, such as polycythemia vera (PV) and essential thrombocythemia (ET) [7]. In a systematic review on the use of thrombophilia testing in patients with MINOCA, factor V Leiden was observed in 12% of these patients, and protein C or S deficiency was observed in 3% of them [51]. Autoimmune antiphospholipid syndrome has been found in almost 7.5% of patients presenting a generic coronary embolism [57]. Instead, thrombotic thrombocytopenic purpura is an infrequent cause of MINOCA, even if its actual weight has not yet been defined [7]. The higher prevalence of thrombophilia in patients with MINOCA should make thrombophilia screening routinary, especially in young fertile women. Furthermore, in the case of a positive result for any thrombophilia, long-term anticoagulant treatment should be considered [56].

Extrinsic vascular compression can be related to myocardial edema, such as in myocarditis. In the acute phase of myocarditis, interstitial edema is relevant in association with inflammatory infiltrate. It can cause considerable extrinsic vascular compression, resulting first in hypoxia and then in myocardial ischemia, by reducing myocardial blood flow, even with only local myocardial damage [58]. The prevalence of myocarditis among patients with a clinical diagnosis of MINOCA is around 33% [58]. The most common cause of biopsy-confirmed myocarditis is a viral infection, mainly sustained by adenoviruses, parvovirus B19, human herpesvirus 6, and coxsackie viruses. In particular, parvovirus B19-related myocarditis can mimic MINOCA [59].

Coronary endothelial dysfunction accounts for up to 60–70% of INOCA and up to 25% of MINOCA. Endothelial dysfunction among these patients is typically expressed according to a coronary microvascular spasm [17,40,52]. To date, it is evident that MINOCA can be linked to microvascular spasm when coronary microcirculation responds exaggeratedly to vasoconstrictive stimuli [49]. This inadequate vasoconstrictive response can be demonstrated by the intracoronary administration of acetylcholine, when this molecule reproduces symptoms and electrocardiogram alterations in patients without epicardial coronary artery diameter change [60]. Microvascular spasm is usually clinically associated with angina at rest, and occasionally, it can lead up to myocardial infarction [53]. Microvascular spasm induced by the intracoronary administration of acetylcholine was reported in almost 50% of patients with stable chest pain and non-obstructive CAD [17]. The endothelium plays a central role in modulating smooth muscle function by releasing vasoactive substances [61,62]. All cardiovascular risk factors and the atherosclerotic process, in the long term, render the vascular endothelium dysfunctional, and the normal vasodilator response to pharmacological and physiological stimuli becomes attenuated both in epicardial arteries and in the coronary microcirculation. It translates into a weak rise and/or reduction of CBF [17,61]. Moreover, functional abnormalities of smooth muscle cells regulating vascular tone in arterioles contribute to CMD. An attenuated vascular smooth muscle relaxation is documented in patients with CMD due to arterial hypertension, dyslipidemia, smoking, obesity, diabetes mellitus, or renal impairment. This has been demonstrated by the use of vasodilator substances, such as adenosine, papaverine, and dipyridamole [17]. 

Microvascular functional abnormalities and structural abnormalities are not independent of each other. For example, a persistent change in microvascular tone results in the development of structural abnormalities: luminal narrowing due to inward remodeling of intramyocardial arterioles, rarefaction of microvessels, and microembolization after the spontaneous or iatrogenic atherosclerotic plaque rupture [52].

To sum up, the role of CMD in INOCA and MINOCA requires further investigations. Unanswered questions remain the role of single factors in causing CMD and whether CMD only causes INOCA and MINOCA or it can also be a consequence of them. Patients with INOCA and MINOCA should also be stratified for their risk of long-term cardiovascular events. In this scenario, the coronary angiography-derived IMR could be used to objectively stratify these patients [44]. 

Additional knowledge is also crucial to achieving patient-specific treatments in INOCA and MINOCA, which certainly include the treatment of cardiovascular risk factors and atherosclerosis. Recent evidence suggests that clinical outcomes will be improved by stratifying antianginal therapies according to the assessment of CMD in these patients [63]. 

## 4. Invasive and Non-Invasive Assessment of Coronary Microvascular Dysfunction

Diagnostic methods of INOCA and MINOCA include both invasive and non-invasive procedures; however, no current technique is able to anatomically visualize the coronary microcirculation in vivo. However, the assessment of the coronary microcirculation can be performed only after having excluded the presence of hemodynamically significant stenosis of the epicardial arteries [64].

As far as the invasive evaluation of the coronary microcirculation is concerned, the COVADIS study released international standardized diagnostic criteria for CMD [65]. Both coronary flow reserve (CFR) and IMR are needed to perform CMD diagnosis. Abnormal CFR is defined as <2.0, while abnormal IMR is defined as ≥25. Namely, microcirculation is studied through the evaluation of the CFR, which represents the relationship between the coronary flux during maximal hyperemia and at rest. In order to evaluate coronary vasomotor dysfunction, the acetylcholine provocation test allows the assessment of endothelial-dependent microvascular function. Intracoronary and/or systemic adenosine infusion allows the assessment of endothelial-independent microvascular function. During hyperemia induced by adenosine, the assessment of the fractional flow reserve (FFR) is also possible in the case of an intermediate atherosclerotic plaque, defined as the ratio of mean coronary pressure distal to stenosis and mean aortic pressure under maximal hyperemia conditions [66]. Furthermore, high values of IMR (≥25), which stands for increased microvascular resistance, and CMD presence also have a prognostic value. When IMR is >40 after primary coronary angioplasty, it is associated with a higher incidence of MACE within 30 days [67], a higher risk of microvascular obstruction [68], and hypercoagulability [69]. It has also been proven to be useful in guiding anti-anginal therapy in patients with angina and non-obstructive CAD [70]. It is an independent predictor for non-fatal myocardial infarction, stroke, HF, re-hospitalization due to angina, and cardiovascular death [44,71]. Moreover, angio-IMR is a new angiographic index, pressure-wire free, initially ideated for the measurement of microcirculatory resistances in patients affected by STEMI [72]. Scarsini et al. demonstrated that it is characterized by a diagnostic accuracy similar to normal IMR, which applies to all coronary syndromes, both acute and chronic [73]. Moreover, in patients affected by STEMI, an angio-IMR value >40 is strongly related to a higher risk of hospitalization for HF and cardiovascular death, compared with a preserved angio-IMR value [74].

The assessment of microvascular function during coronary angiogram may be carried out through Doppler flow velocity and coronary bolus thermodilution [75]. CFR, for instance, is assessed through the thermodilution method. It consists of calculating the average mean transit time of a saline bolus injected into the coronary artery, administered at room temperature, mixed with blood at body temperature, and dividing the hyperemic mean transit time by resting mean transit time [76]. IMR is calculated as the product of distal coronary pressure at maximal hyperemia, then multiplied by the hyperemic mean transit time [77]. The Doppler method, instead, involves a Doppler crystal that measures the average peak velocity (AVP), providing the CFR by the ratio between the AVP value during hyperemia and under resting conditions. Both methods have different weaknesses and technical disadvantages, with increased interobserver variability in the Doppler method, whereas the thermodilution method is more feasible but characterized by higher intraobserver variability [75,78]. 

The non-invasive evaluation of microcirculation is performed after ruling out hemodynamically significant epicardial stenosis. It can be performed through coronary computed tomography (CT). The non-invasive evaluation of microcirculation relies on positron emission tomography (PET), transthoracic color Doppler echocardiography, perfusion cardiac magnetic resonance (CMR), and perfusion coronary CT. PET, whose measures have been widely validated, represents the most accurate technique. It allows CFR assessment by measuring the blood flow difference to the myocardium at rest and under pharmacological stress [79]. The accuracy and reproducibility of PET for the quantitative measurement of myocardial blood flow and CFR have been widely validated both in humans and in animals. The typical protocol of evaluation consists of a rest myocardial perfusion study and a vasodilation-stress study based on the use of a radiotracer, allowing the quantification of both regional and global myocardial blood flow to obtain CFR [17,80]. 

Transthoracic color Doppler echocardiography allows studying the coronary flow velocity reserve (CFVR) by means of the pulsed-wave Doppler technique. CFVR is the ratio of the diastolic peak velocity of the CBF at rest and after maximal hyperemia. A CFVR ≤ 2–2.5 defines CMD. This technique samples the anterior descending coronary artery and uses it as a reference for the whole coronary microcirculation [81]. Schroder et al. demonstrated that the CFVR value has a prognostic value [82]. The Doppler technique represents a low-cost option free of ionizing radiation, even if it demands the challenging visualization of the proximal coronary arteries. 

CMR, through the injection of a gadolinium-based contrast agent and a perfusion study, can be used to quantify myocardial perfusion because it enables the evaluation of the regional and global myocardial perfusion reserve. Perfusion is directly proportional to T1 signal intensity, given by gadolinium diffusion from the microcirculation to the interstitial space [83,84]. The usual imaging protocol is based on a rest myocardial perfusion study and a vasodilator stress myocardial perfusion study, following the injection of a gadolinium-based contrast agent; the regional and global myocardial perfusion is obtained by using semiquantitative or quantitative models (i.e., CFR). The main advantages are great spatial resolution, transmural characterization of blood flow, absence of radiation, and global assessment of cardiac function and structure. Moreover, the CFR evaluation and the myocardial perfusion reserve index have been demonstrated to predict the rate of MACE with high prognostic power beyond the presence of late gadolinium enhancement and ischemia [17,80,85]. 

Lastly, dynamic CT scanning can produce estimates of absolute myocardial flow through models previously used for CMR, creating the chance to detect anatomical and functional abnormalities of the myocardium and the coronary circulation during the same examination.

## 5. MINOCA and Coronary Microvascular Dysfunction: The Genetic Susceptibility

The study of the genetic mechanisms underlying the pathophysiology of cardiovascular diseases is obtaining increasing interest. Beyond traditional cardiovascular risk factors, a role as primum movens for genetic susceptibility has been hypothesized in determining IHD. It is known that more than 50% of the susceptibility to IHD depends on genetic variants [86], mostly consisting of single-nucleotide polymorphisms (SNPs). They have been identified through genome-wide association studies (GWAS) [87]. Genetic variants may predispose, particularly affecting proteins involved in CBF regulation, to CMD and, therefore to INOCA and MINOCA. This is associated with CBF imbalance and myocardial ischemia. It is important to notice that genetics may represent not only a predisposing but also a protective determinant for myocardial ischemia. Despite this, several multiple gene loci associated with the development of CAD have been identified, and few results have been obtained for understanding the genetic mechanisms underlying microcirculatory dysfunction.

Recently, molecular pathways associated with an increased risk of CFR alterations have been identified in intronic sequences of the vascular endothelial growth factor-A (VEGF-A) and cyclin-dependent kinase inhibitor 2B-AS1 (CDKN2B-AS1) genes [88]. The first GWAS [89] attributed significant relevance to a locus on chromosome 9p21, CDKN2B-AS, a long non-coding antisense ribonucleic acid (RNA), also called antisense non-coding RNA in the INK4 locus (ANRIL) [89]. It is expressed both in VSMCs and endothelial cells of the coronary arteries. Its deficiency causes abnormal cell proliferation and senescence with repercussions on the coronary microcirculation. In addition, GWAS have highlighted the interaction between this locus and inflammatory mediators and/or lymphoblastoid cell growth, such as interferon γ (IFN γ). Therefore, the relevant interchanging role played by inflammation in the pathophysiology of IHD has been confirmed [90].

VEGF-A is a chemotactic and mitogenic agent for endothelial cells. It regulates their proliferation and migration during the process of coronary morphogenesis. Furthermore, it constantly affects the functionality of the endothelium during vascular regeneration [91]. Its decreased expression in some genetic variants is correlated with vascular dysfunction and lower survival of endothelial cells, due to apoptotic processes and repairing mechanisms abnormalities [88].

Other findings concern hemeoxygenase1 (HMOX1) promoter polymorphisms, a stress-induced enzyme with a protective role against myocardial ischemia, including ischemic injury. HMOX1 catalyzes the degradation of heme to iron, carbon monoxide, and biliverdin. An association has been shown among SNPs with long HMOX1 promoter guanine-thymine repeats, cardiovascular diseases, and a reduced left ventricular ejection fraction [92]. Furthermore, how HMOX1 confers protection against ischemia injury has to be fully understood in an animal study [93].

Another decisive factor in genetic predisposition to microvascular dysfunction is ET-1. Acting as the natural counterpart of the vasodilator NO, ET-1 is a powerful vasoconstrictor. Binding the endothelin-A (ET-A) and endothelin-B (ET-B) receptors, it affects vascular tone and proliferation. Excess ET-1, activating the G-protein-coupled ET-A receptor in VSMCs, induces vasoproliferative effects, endothelial dysfunction, and inflammation [94]. Recently, it has been demonstrated that a gene locus on chromosome 6p24 (PHACTR1/EDN1) regulates ET-1 gene expression and that the allelic variant rs9349379-G is associated with the increased plasma concentrations of ET-1. Consequently, the risk of atherosclerotic epicardial coronary heart disease, myocardial infarction, and CMD increases [95]. It has already been shown that higher plasma concentrations of ET-1 in patients with microvascular angina are related to an increase in coronary vascular resistance and impaired CBF [96]. Ex vivo studies have confirmed that subjects with functional allele rs9349379-G respond to ET-A receptor blockade, such as zibotentan, an orally active ET-A receptor antagonist [97]. However, results from future trials are needed to determine whether patients with microvascular angina represent new potential beneficiaries of ET-A antagonist therapy.

Given the known importance of the regulatory role of NO and ion channel on vascular tone, and considering that allelic variants of eNOS, such as rs1799983_G/T, are independent predictors of IHD, it is not surprising that genetic alterations of these predispose also to IHD, affecting both epicardial arteries and microcirculation [98]. NO activity is mediated by cyclic guanosine monophosphate (cGMP) and guanosine-5’-triphosphate (GTP), synthesized through guanylyl cyclase soluble and resulting in the activation of K-ATP channels. K-ATP channels consist of the subunit Kir6.1 and/or Kir6.2 and sulfonylurea-binding subunits (SUR1, 2A, or 2B). It has been demonstrated that genetic variants of the potassium inwardly rectifying channel subfamily J member 11 (KCNJ11) gene, coding for Kir6.2, may produce alterations in K-ATP channel function, impacting myocardial ischemia susceptibility [99]. Other SNPs may be protective against CMD and IHD [27,98,100]. For example, the rs5215_G/G polymorphism of KCNJ11 has demonstrated a protective role against IHD [100]. In addition, the co-presence of the SNPs rs5215_G/G of KCNJ11 and rs1799983_T/T of the nitric oxide synthase 3 (NOS3) gene may play a protective role against myocardial ischemia [101]. This allows the hypothesis of cross-talk between the NO pathway and K-ATP in coronary circulation [101]. Additionally, other ion channels, in particular Kv1.3 and Kv1.5, have been demonstrated as crucial mediators between CBF and myocardial metabolism [102,103].

Gender differences in SNPs are currently under analysis. In fact, it is now recognized that males and females are exposed, during development and aging, to different hormonal changes and risk factors [104] and that they show different vessel diseases [105]. Male sex is related to a higher prevalence of extensive and obstructive epicardial lesions compared to the female gender. Female sex shows a higher prevalence of microcirculatory dysfunction [106]. Furthermore, females with anginal symptoms and microcirculatory dysfunction are three times more likely to experience MACE than women without symptoms and IHD. In the male group, no differences were found between patients with microvascular angina and asymptomatic patients without CAD. This demonstrates how microcirculatory dysfunction affects and determines a different prognosis between men and women [107]. Currently, no association between SNPs and an increased risk of microcirculatory disease has been identified in females, while three genetic variants involving three proteins have been identified in men: 5’-nucleotidase ecto (NT5E), myosin heavy chain 15 (MYH15), and VEGF-A [88]. The variant gene for NT5E is associated with reduced CFR and increased coronary calcification [88,108]. Genetic variants of MYH15, encoding myosin heavy-chain polypeptide and regulating vascular tone, have been associated with an increased risk of CMD; however, their role in the pathogenesis of microcirculatory dysfunction needs further studies.

MINOCA is also a non-rare complication in hematologic diseases based on specific genetic mutations, particularly in chronic myeloproliferative Philadelphia-negative neoplasms (cMPNs) [109,110,111]. In these patients, thrombotic events are the leading cause of death, with a much higher prevalence compared to the general population. Even if the pathogenesis of endothelial dysfunction in cMPNs remains unclear, shear stress, blood hyperviscosity, and hypoxemia seem to play a primary pathophysiological role. As far as genetic mutations are concerned, the most explored is the Janus kinase 2 (JAK2) V617F mutation. It determines an acquired gain-of-function mutation in exon 14 of the gene, present in 75% of cMPN cases. Pósfai et al. examined this correlation both in ET and PV: JAK2 V617F mutation positivity was present in 71.4% of myocardial infarctions, among which 21.4% have been represented by MINOCA [109]. Furthermore, ET and PV are characterized by altered levels of NO derivatives. Hydroxyurea treatment may lead to higher values of NO derivatives, with a significant additional antithrombotic mechanism [110]. In addition, genetic mutations, in particular the V617F mutation of JAK2 in MPN patients, may be associated with endothelial dysfunction and coronary spasm [111]. 

The main genetic variants discussed in this review and associated with microvascular dysfunction are summarized in Table 1.

Future studies are essential in order to gain a deeper understanding of the role of multiple genetic variants, mostly still unknown, in the genesis of microcirculation dysfunction. Progress in research would allow the early identification of high-risk patients and the development of pharmacological, patient-tailored strategies.

## 6. Conclusions

MINOCA represents a relevant cause of AMI, particularly common in certain groups of patients. In myocardial infarction, due to atherosclerotic disease of epicardial arteries, the triggering cause may be easily identified and treated with coronary revascularization. Moreover, cardiovascular risk factors may be strictly controlled through pharmacological therapies. Conversely, the pathophysiological mechanisms of MINOCA often remain unidentified, and risk factors are often unknown, exposing patients to recurrences, adverse events, and low quality of life. Moreover, the heterogeneity of MINOCA pathophysiological mechanisms makes the clinical management of these patients particularly challenging. In this review, we focused on the role of CMD because it has a central role in the complex pathophysiology of MINOCA and INOCA. The assessment of coronary microcirculation may be performed invasively and non-invasively, and it should be implemented both in MINOCA/INOCA and in myocardial ischemia due to atherosclerotic disease, also because of the main pathophysiological role of CMD in patients undergoing percutaneous coronary interventions [112,113]. CMD consists of structural and functional alterations of microcirculation, which hamper the cross-talk between coronary circulation and myocardial metabolism. In this scenario, genetic susceptibility may have a primary role, affecting proteins involved in the regulation of vasomotor tone, endothelial function, cell proliferation, and atherosclerotic plaque stability. Further identification of genetic variants associated with CMD may improve the actual knowledge regarding the pathophysiology of MINOCA, also offering possible innovative targets for the management of these patients. 

## Figures and Tables

**Table 1:** Summary table of proteins and genetic variants associated with coronary microvascular dysfunction.

Protein	Pathophysiological Mechanism	Reference
**VEGF-A**	Reduced expression → Repairing mechanisms abnormalities and increased apoptotic process → VSMCs and endothelial cells dysfunction → CMD	[88]
**CDKN2B-AS1**	Deficiency → abnormalities in VSMCs and endothelial cells proliferation and senescence → CMD	[88,89]
**HMOX**	SNPs → reduced protection against ischemic injury → CMD	[92,93]
**ET-1**	SNP rs9349379-G → increased plasma concentration of ET-1 → vasomotor tone impairment and atherosclerotic disease progression → CAD and CMD	[94,95,96,97]
**eNOS**	SNP rs1799983_G/T → substitution of guanine with thymine with consequent aminoacidic change from glutamic acid to aspartic acid → lower mRNA levels → reduction in eNOS expression → endothelial dysfunction → CAD and CMD	[98,99]
**K-ATP**	SNP rs5215_G/G of KCNJ11 → valine–isoleucine substitution → K-ATP gain of function → increased vasodilation and shear stress reduction.	[100,101]
**NT5E**	Genetic variants → CFR reduction and increased coronary calcification	[88,108]
**MYH15**	Deregulation of vascular tone → increased risk of CMD	[88]
**JAK2**	V617F mutation → endothelial dysfunction and coronary spasm	[109,110,111]

VEGF-A: Vascular endothelial growth factor-A; CDKN2B-AS1: Cyclin-dependent kinase inhibitor 2B-AS1; HMOX: Heme oxygenase; ET-1: Endothelin-1; eNOS: Endothelial nitric oxide synthase; K-ATP: ATP-sensitive potassium; NT5E: 5′-Nucleotidase ecto; MYH15: Myosin heavy chain 15; VSMCs: Vascular smooth muscle cells; CMD: Coronary microvascular dysfunction; CAD: Coronary artery disease; SNPs: Single-nucleotide polymorphisms; KCNJ11: Potassium inwardly rectifying channel subfamily J member 11; CFR: Coronary flow reserve; JAK2: Janus kinase 2.

## Data Availability

Not applicable.
